# Phylogenetic Analysis of Indian Dromedary Breeds Based on the Mitochondrial D-Loop Marker

**DOI:** 10.3390/ani15213070

**Published:** 2025-10-23

**Authors:** Sagar Ashok Khulape, Carlos Iglesias Pastrana, Ratan Kumar Choudhary, Shyam Sundar Choudhary, Rakesh Ranjan, Kashi Nath, Rakesh Kumar Poonia, Samar Kumar Ghorui, Anil Kumar Puniya

**Affiliations:** 1 ICAR-National Research Centre on Camel, Bikaner 334001, Rajasthan, India; vetdrrkc@gmail.com (R.K.C.); shyamuniverse@gmail.com (S.S.C.); ranjanpau@gmail.com (R.R.); drkasinth@gmail.com (K.N.); rpoonia25@gmail.com (R.K.P.); ghorui92@gmail.com (S.K.G.); akpuniya@gmail.com (A.K.P.); 2 The Andalusian Institute for Agricultural, Fishing, Food and Ecological Production Research and Training (IFAPA), Centro Alameda del Obispo, Alameda del Obispo, 14004 Cordoba, Spain

**Keywords:** dromedary camel, mitochondrial D-loop, genetic uniformity, conservation, phylogenetic analysis

## Abstract

**Simple Summary:**

Dromedary camels remained to be an important resource in arid and semi-arid livestock keeping in India. Still, a declining trend in dromedary population is observed recorded in previous decade. In this study, the mitochondrial D-loop region of nine Indian dromedary breeds was compared those of Arabian and Iranian populations. The study highlights limited genetic variation and common maternal ancestry in D-loop region for Indian dromedary.

**Abstract:**

The mitochondrial displacement loop (D-loop) region is a non-coding control region that plays a crucial role in replication and transcription, serving as an informative marker for evolutionary and demographic studies. In this study, the complete mitochondrial D-loop sequences from NCBI public database were analyzed across nine Indian and other dromedary populations. Evolutionary and pairwise sequence analysis indicate distinct separation from foreign populations and substantive clustering of Indian breeds within a monophyletic clade. Indian breeds showed greater than 99.4% sequence identity, minimal diversity (π ≈ 0.003), and limited divergence (k = 3–4), whereas Arabian and Iranian populations exhibited more prominent variability (π ≈ 0.004–0.0044; k ≈ 5). Nucleotide composition analyses corroborated the AT-rich nature of the D-loop with conserved sequence length and enrichment of CpG motifs. This suggests selective conservation of functional elements in the D-loop sequence region. Correlation and correspondence analyses highlighted non-random nucleotide usage and repeat dynamics consistent with replication-associated mutational pressures. Demographic structural diversity showed that nearly all genetic variation was distributed among populations (~99.9%), with minimal variation within breeds. Pairwise differentiation values indicated substantial divergence between Indian and foreign breeds, with Indian desert breeds displaying restricted differentiation, possibly due to shared maternal ancestry. Neutrality test results for the sequence dataset interpreted ongoing demographic expansion or bottleneck recovery for the Arabian, Iranian, Sindhi, and Kharai populations. In contrast, for other Indian desert breeds, the neutrality test values that were closing towards zero may express current population shrinkage. Conserved transcription factor binding motifs further support the role of purifying selection on sequence functional constraints. These findings highlight that Indian dromedaries bear highly conserved mitochondrial D-loop sequences, which are influenced by purifying selection and demographic stability. This low mitochondrial diversity in non-coding sequence can mirror the declining population size and emphasizes the urgent need for targeted conservation strategies.

## 1. Introduction

Mitochondrial DNA (mtDNA) has been used as a valuable molecular marker for investigating the evolutionary dynamics, population structure, and domestication history of animals [1]. The distinctive characteristics of mitochondrial DNA (mtDNA)—primarily its maternal inheritance with only rare paternal contribution, absence of recombination, and highly compact genetic organization—make it particularly well-suited for phylogenetic and population genetic analyses [2,3,4]. Within the mitochondrial genome, the displacement loop (D-loop), a non-coding region, is the primary control site for replication and transcription initiation [5]. The D-loop consists of regulatory elements and exhibits substantial sequence variation, offering a window into evolutionary processes at both functional and structural levels [6].

Across domesticated species, mtDNA D-loop analysis has provided valuable insights into maternal lineages, domestication processes, and evolutionary adaptation. For cattle, full mitogenome studies have helped delineate multiple hot spots of domestication and introgression events [7]. Similar studies in sheep (*Ovis aries*) and goats (*Capra hircus*) have illustrated how mitochondrial markers reflect the geographic expansion and climatic adaptation of ancient livestock populations [8,9]. In camelids, recent whole mitogenome studies have revealed signatures of purifying selection and demographic expansion, highlighting the evolutionary importance of the mitochondrial genome. Bahbahani et al. [10] identified both pervasive purifying selection and localized signals of positive selection on dromedary mtDNA, while Mohandesan et al. [11] reported strong purifying selection and long-term divergence between wild and domestic Bactrian camels. In addition, molecular studies using ATP6/ATP8 genes or partial D-loop segments have elucidated species divergence between Old World camelids (*Camelus dromedarius*, *Camelus bactrianus*, and *Camelus ferus*) and New World camelids (*Lama glama*, *Vicugna vi-cugna*, *Lama guanicoe*, and *Lama pacos*) [11,12]. Together, these findings underscore the role of selective forces in shaping camelid mitochondrial diversity and helped clarify domestication events and hybridization patterns.

However, compared to cattle and small ruminants, camelid mitochondrial studies remain relatively limited, and comprehensive analyses of complete D-loop sequences are scarce [13]. This limitation restricts our understanding of structural motifs, nucleotide usage biases, and repeat dynamics, which are critical for replication fidelity, mutational processes, and evolutionary stability [14,15].

The dromedary breeds in India represent a unique component of camelid genetic resources, adapted to arid and semi-arid environments. These breeds are historically engendered by natural events and anthropogenic selection practices [16,17]. Despite their ecological and economic importance, Indian camel populations have experienced a steady demographic decline, underscoring the urgency for conservation-oriented genetic studies [16]. Yet, their mitochondrial diversity and demographic history remain poorly characterized, with most available studies restricted to partial sequences or a few coding genes. Indian dromedary breeds—including Bikaneri, Jaisalmeri, Jalori, Kharai, Kutchi, Malvi, Marwari, Mewari, and Sindhi—have not been comprehensively studied using the complete mitochondrial D-loop.

Additionally, the role of evolutionary pressures not only on the primary sequence but also on structural motifs, nucleotide usage biases, and repeat dynamics [15]. Thus, focusing only on coding genes or limited haplotype sets can mask the broader structural, compositional, and spatial dynamics critical to understanding evolutionary pressures and functional constraints acting on the mitochondrial genome. Thus, a compositional and spatial analysis of D-loop sequences can complement conventional methods and offer a deeper insight into mutation hotspots and functional constraints. The low mitochondrial divergence among Indian dromedary breeds has been reported for ATP8 and ATP6 by other investigators [18]. In contrast, for the camel populations from the Arabian Peninsula, a higher haplotype diversity and phylogeographic structuring have been observed [11,14]. Similarly, other domestic species in India, such as *Bos indicus*, have demonstrated greater mitochondrial diversification, indicative of multiple domestication events or wider breed structuring [7,8]. In comparison, Indian camels present an inconspicuous case of genetic uniformity. While reflecting breeding and maternal continuity, this homogeneity raises concerns about the potential vulnerability of the Indian dromedary to population decline. Moreover, this reduced genetic diversity can impact the adaptability of the species to emerging environmental and disease challenges, particularly under scenarios of climate change and habitat disruption [17,19].

At the molecular level, comparative studies have identified conserved structural domains crucial for replication and transcription regulation, despite environmental extremes in the D-loop sequence [1,15,16]. Hence, the structural and functional insights from non-coding mitochondrial regions, such as the D-loop, will enhance our understanding of livestock adaptation, resilience, and evolutionary trajectories.

The present study addresses this gap by performing a detailed compositional and evolutionary analysis of the mitochondrial D-loop across Indian dromedary breeds. By integrating sequence identity, dinucleotide abundance, nucleotide repeat distribution, phylogenetic reconstruction, and multivariate analyses, this work provides a holistic view of the organization and variability of the D-loop. Such an approach complements conventional haplotype-based analyses and con-tributes to a deeper understanding of demographic processes, evolutionary constraints, and conservation needs of Indian dromedary populations.

## 2. Materials and Methods

### 2.1. Sequence Dataset

India has nine registered dromedary camel breeds, which have diverse geographic distributions and are adapted to various arid and semi-arid environments [20]. The complete Mitochondrial and D-loop nucleotide sequences of Indian dromedary camel breeds—Bikaneri, Jaisalmeri, Jalori, Kharai, Kutchi, Malvi, Marwari, Mewari, and Sindhi—already reported at NCBI GenBank database were retrieved (Supplementary Table S1). The representative sequences of dromedaries from the Arabian Peninsula and Iran were also included for comparative evolutionary analysis. The wild camel mtDNA D-loop sequence was used as an outgroup to root the tree.

### 2.2. Evolutionary Distance and Phylogenetic Analysis

The percent nucleotide identity among D-loop sequences of Indian dromedary breeds was computed using the MegAlign™ module from the Lasergene software suite (DNASTAR, Madison, WI, USA, version 17.4, 2024). Multiple sequence alignment of the sequence dataset was done using the Clustal W algorithm with default parameters in MEGA version 12.0.14 [21]. The phylogenetic relationships were inferred using a Bayesian approach with the default parameters in the BEAST software (v1.10.4) [22]. The posterior distribution of trees obtained from BEAST was summarized into a Maximum Clade Credibility (MCC) tree using TreeAnnotator (v2.6.7) for the dataset, with the initial 10% of trees discarded as burn-in. The MCC tree represents the topology with the highest product of posterior clade probabilities.

### 2.3. Compositional Analysis

The mitochondrial D-loop sequence dataset for the Indian dromedary breeds was analyzed for sequence composition. Basic sequence parameters such as sequence length and nucleotide composition percentages (%A, %T, %G, %C, %A + T, and %G + C) were calculated using Microsoft Excel. The analyses of mononucleotide repeats (A, T, G, C), dinucleotide frequencies (%AA, %AT, %AC, %AG, %TA, %TT, %TC, %TG, %CA, %CT, %CC, %CG, %GA, %GT, %GC, %GG), and nucleotide distance distribution were performed using the Nfeature webserver [23]. The relative abundance of the CpG motif in the sequence dataset was calculated from the frequency of CpG and GpC dinucleotide.

### 2.4. Correlation Analysis

Pearson correlation coefficients were computed among nucleotide composition parameters using PAST software (v4.13) [24] to examine inter-relationships. The significance level was set at *p* < 0.05 and *p* < 0.01 for analysis.

### 2.5. Correspondence Analysis

A correspondence analysis was performed for the sequence dataset to identify trends in nucleotide composition and usage patterns in the mitochondrial D-loop region. The frequency distribution for mononucleotide repeats and nucleotide occurrence patterns were analyzed separately. This multivariate approach enables visualization of the variation in repeat frequency and relative positional distribution of each nucleotide across the sequence dataset. The statistical analysis was executed using the SAS 9.4 software.

### 2.6. Population Structure Analysis

The analysis for mitochondrial diversity in dromedary breeds, with respect to the number of variable sites (S), number of haplotypes, haplotype diversity (Hd), nucleotide diversity (π), and the average number of nucleotide differences (k) for the dataset, was performed using DNASP version 6 [25]. The association among observed haplotypes in the nucleotide dataset was examined through the construction of a Median-Joining network implemented in PopART (Population Analysis with Reticulate Trees) [26]. Analysis of molecular variance (AMOVA) was conducted within and between population groups using Arlequin version 3.5 [27]. Pairwise neutrality tests, including Fu’s Fs [28] and Tajima’s D [29], were also estimated using Arlequin v3.5 using default settings.

### 2.7. Prediction of Transcription Factor Binding Sites

Transcription factor binding site (TFBS) prediction was performed using the TFinder web tool (https://tfinder-ipmc.streamlit.app/; accessed on 21 July 2025), which utilizes experimentally validated transcription factor motifs for binding site identification. The nucleotide sequence dataset was submitted in FASTA format to the TFinder platform. Default parameters were used for the analysis, which includes scanning for putative TFBSs based on sequence similarity scores and computing relative binding affinity scores. To ensure reliability, predicted binding sites with high relative scores (above 0.75) and statistically significant *p*-values (below 0.01) were considered for downstream interpretation.

## 3. Results

### 3.1. Phylogenetic Relationship and Sequence Identity

**Figure 1:**
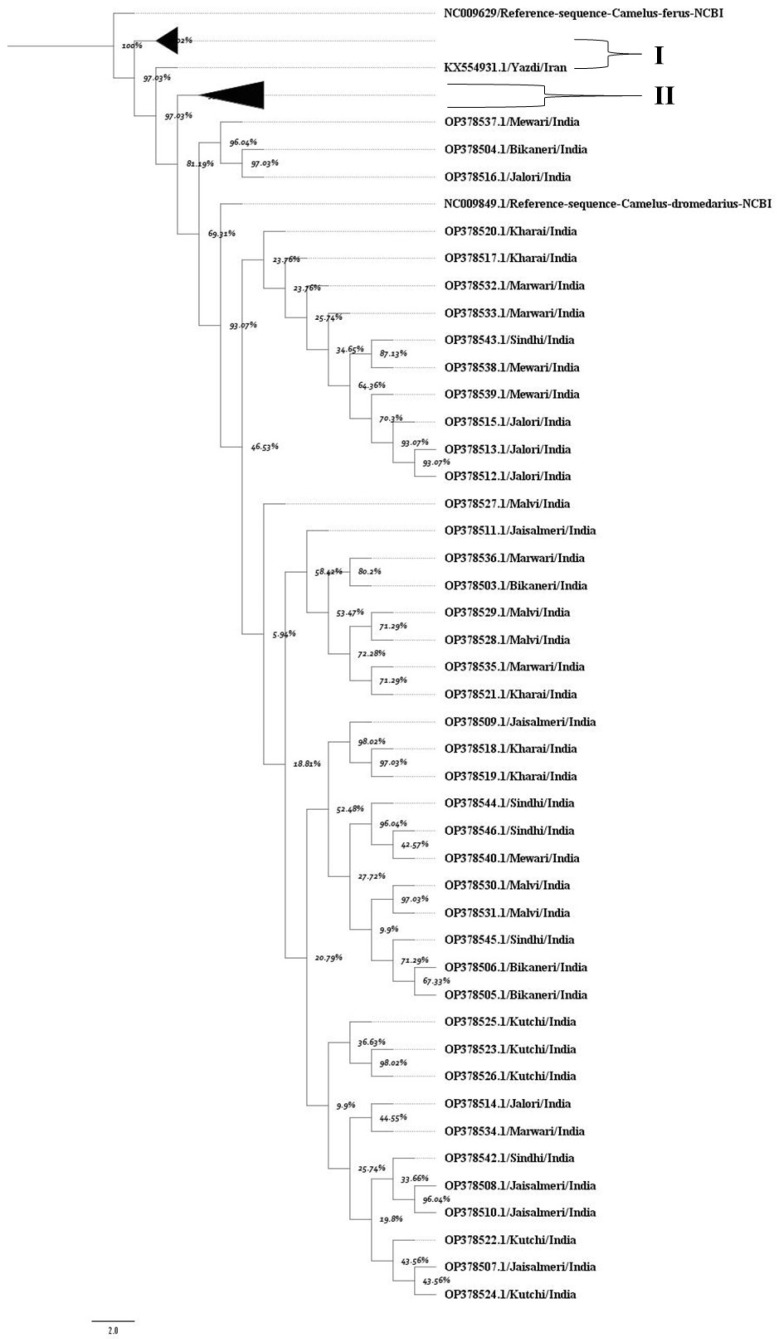
The maximum clade credibility tree indicates the phylogenetic relationship of the mitochondrial D loop dataset. Each node represents percent posterior probability values. The nodes corresponding to D-loop sequences from (I) Iran and (II) the Arabian Peninsula were collapsed for better visualization of the tree. The scale bar indicates the branch length. NCBI reference sequences for the D-loop region of *Camelus ferus* and *Camelus dromedarius* were incorporated to enhance the reliability of the tree.

The MCC tree confirmed a high degree of genetic similarity among Indian dromedary breeds (Figure 1). A principal bifurcation was observed between the dromedary and wild camel populations. Indian dromedary breeds form a closely clustered, separate, and well-supported monophyletic clade, indicating their shared evolutionary ancestry and closer mitochondrial similarity. Additionally, the posterior probability values of each node supported the close evolutionary relationship among Indian breeds. The nucleotide sequences of dromedary breeds from Iran, as well as those from the Arabian Peninsula, formed a separate monophyletic group. The high posterior probability support at most of the nodes in the tree confirms the robustness of these evolutionary relationships.

The pairwise sequence identity analysis of the mitochondrial D-loop region among Indian dromedary breeds revealed high genetic similarity. The within-breed identity ranged from 99.32% (Malvi) to 99.9% (Mewari), indicating minimal within-breed variation. The between-breed identity values were also consistently high, typically exceeding 99.5%, with the lowest observed between Malvi and other breeds (e.g., 99.49–99.55%) (Table 1).

Notably, the highest between-breed identity was observed for the Mewari and Sindhi (99.92%), followed closely by Mewari–Marwari (99.9%) and Marwari–Sindhi (99.89%) camels. These values suggest a close mitochondrial lineage and possibly shared maternal ancestry among these breeds. On the other hand, like Malvi and Jalori displayed slightly lower pairwise identities, though still within the high similarity threshold (>99.4%).

### 3.2. Nucleotide Composition

The mitochondrial D-loop region exhibited a consistent sequence length of approximately 1213 nucleotides, with minor variation noted in Malvi (1207–1213 nt) and Kharai (1211–1213 nt) (Table 2). The D-loop region remained AT-rich, with mean A + T content ranging from 52.98% to 53.39%, while G + C content varied between 46.61% and 47.02%. Among nucleotides, adenine (A; 29.65–29.72%) and cytosine (C; 29.30–29.78%) were the most abundant, followed by thymine (T; 23.33–23.71%) and guanine (G; 17.18–17.35%). Notably, the D-loop exhibited lower A + T and higher G + C content compared to the complete mitochondrial genome. The whole mitochondrial genome was even more AT-biased, with A- (~30.83%) and T- (~27.10%) as the dominant bases and relatively lower C- (~26.59%) and G- (~15.49%) proportions. This compositional diversity of the D-loop reflects the functional and evolutionary uniqueness and shows a distinct base usage trend compared to the rest of the mitochondrial genome. Minor standard deviations (SD) observed across nucleotide percentages also indicate substantial sequence conservation within and among breeds for both D-loop and whole mitochondrial genomes (Table 2).

### 3.3. Dinucleotide Abundance Patterns

**Figure 2:**
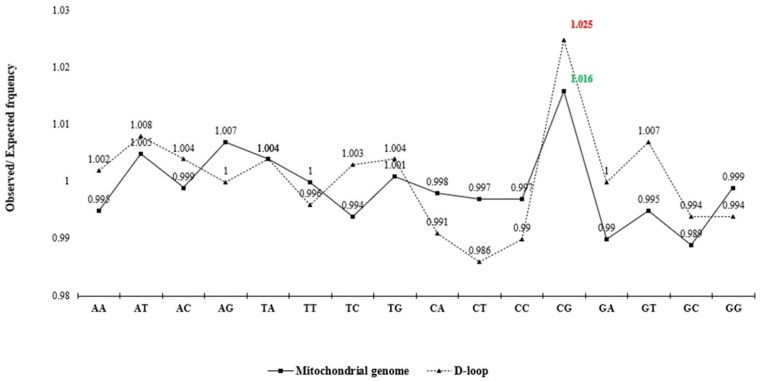
Analysis of relative dinucleotide abundance in mitochondrial genome and D-loop sequence dataset. The line chart shows the mean observed/expected (O/E) frequency ratio for 16 dinucleotides.

**Figure 3:**
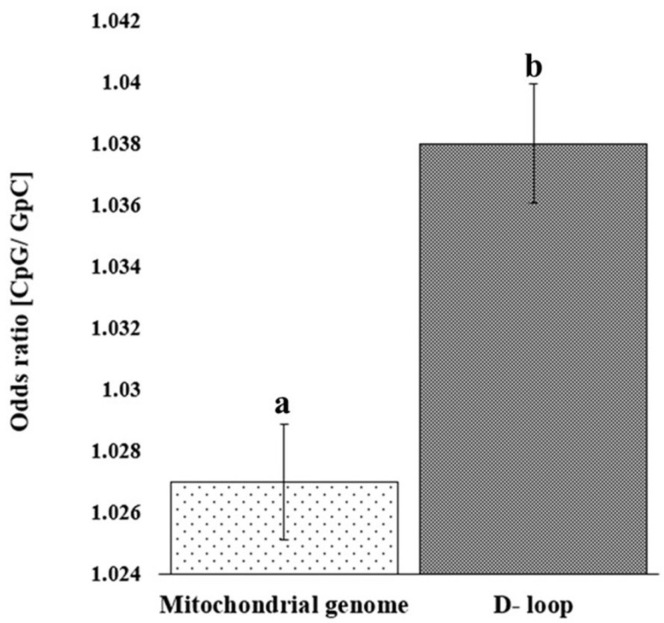
Bar chart depicting the relative abundance of CpG in the D-loop sequence and mitochondrial genome. Error bar showing standard deviation. Bars with different superscripts differ significantly (*p* < 0.05).

The analysis of observed/expected (O/E) dinucleotide frequency ratios across the mitochondrial genome and D-loop region revealed a variable distribution of 16 possible dinucleotide combinations (Figure 2). The O/E ratio provides insight into the relative abundance of dinucleotides beyond simple base composition, offering clues to evolutionary pressures and sequence constraints. Among the 16 possible dinucleotides, CpG motifs displayed a markedly higher O/E ratio in the D-loop region compared to the complete mitochondrial genome. Furthermore, a statistically significant increase (*p* < 0.05) in relative abundance of CpG in the D-loop, compared to the whole mitochondrial genome, was confirmed (Figure 3). This enrichment might reflect evolutionary retention of specific motifs crucial for replication or transcription initiation, particularly within the D-loop where such regulatory elements are typically concentrated.

### 3.4. Correlation Analysis

Correlation analysis revealed several statistically significant associations among the nucleotide composition variables of the mitochondrial D loop (Table 3). A strong negative correlation was observed between %A and %T (r = −0.792, *p* < 0.01), and a positive correlation between %A and %C (r = 0.793, *p* < 0.01), indicating an inverse distribution trend between A and T, and a tendency for co-occurrence between A and C. Similarly, %G showed a negative correlation with %C (r = −0.593, *p* < 0.01) and %A (r = −0.795, *p* < 0.01), suggesting compositional biases in purine and pyrimidine distribution. The complementary balance between AT-rich and GC-rich regions showed perfect negative correlation (r = −1.000, *p* < 0.01).

These base composition patterns were further correlated with nucleotide distance distribution and occurrence of nucleotide repeat. As for Adenine distance distribution (A-DD), it showed a strong positive correlation with %T (r = 0.955, *p* < 0.01) and a negative correlation with %C (r = −0.861, *p* < 0.01), reflecting the influence of adenine on spatial arrangement. Similarly, Adenine repeat frequency was significantly correlated with AT% (r = 0.858, *p* < 0.01), indicating a strong association between AT-richness and homopolymeric adenine stretches. Likewise, trends were seen in nucleotide repeat frequency for Guanine and Cytosine, which exhibited notable correlations with compositional and spatial distribution parameters.

### 3.5. Correspondence Analysis

**Figure 4:**
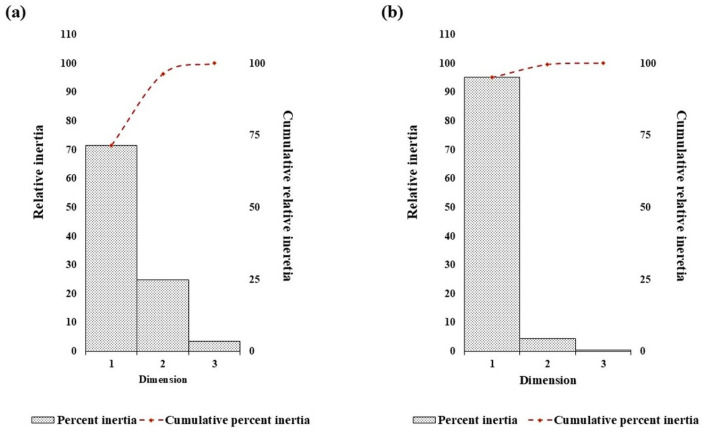
The trend of percent relative and cumulative inertia on the nucleotide composition in the dromedary D-loop sequence with respect to (**a**) nucleotide repeats and (**b**) distance distribution by correspondence analysis.

**Figure 5:**
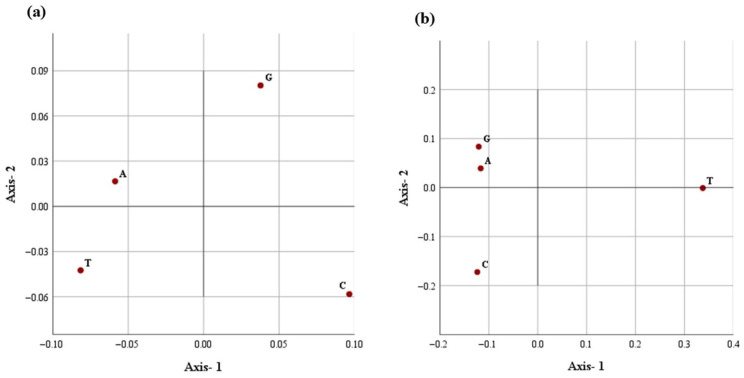
The distribution of each nucleotide in the first two main dimensional coordinates with respect to (**a**) nucleotide repeats and (**b**) distance distribution in the D-loop.

Correspondence analysis was done to interpret underlying trends in nucleotide repeat usage and spatial distribution within the D loop sequences of camel. The analysis of nucleotide repeat frequencies is visualized in Figure 4a and Figure 5a. The first two dimensions explained 96.4% of the total variation, with Axis-1 accounting for 71.5%, indicating a single dominant trend in repeat frequency among nucleotides. This strong dimensionality suggests strong purifying selection and low genetic divergence observed in the mitochondrial D-loop region among Indian dromedary breeds.

A similar correspondence pattern was observed for the spatial distribution of all four nucleotides (Figure 4b and Figure 5b). The G- and C- appear to spread along the primary axis, reflecting their higher variability in repeat frequency. Conversely, A- and T-nucleotides showed a tendency of close clustering, suggesting a more uniform repeat occurrence across breeds. Axis-1 accounted for a higher variation (95.2%), with Axis-2 contributing 4.4%, explaining 99.6% of the total inertia. The analysis indicates a dominant and consistent trend of nucleotide placement across the mitochondrial D-loop in different Indian dromeadry breeds.

### 3.6. Population Diversity Estimates

AMOVA revealed that mitochondrial D loop genetic variation was almost entirely distributed among populations (~99.9%), with only 0.1% attributable to within-population diversity, and this differentiation was statistically significant (*p* = 0.001) (Supplementary Table S2). Pairwise molecular distance confirmed the strong structuring of the hierarchical clustering, showing very high divergence between Iranian/Arabian Peninsula camels and Indian breeds (Supplementary Figure S1). In contrast, Indian desert populations, such as Bikaneri, Jaisalmeri, Marwari, Jalori, and Mewari, were genetically similar, with near-zero molecular distance. Moderate differentiation was observed in Kharai, Sindhi, and Malvi populations. The Arabian Peninsula camel population was highly distinct and comparable in divergence to Iranian camels.

Tajima’s D analysis for Sindhi and Kharai camels exhibited significantly negative Tajima’s D values, reflecting expansion or recovery from past bottlenecks. In contrast, desert Indian breeds showed values close to neutrality, suggesting continuous demographic stability. These results underscore strong genetic structuring (Supplementary Table S2). Further, mtDNA d-loop sequence analysis revealed notable differences in haplotype composition and nucleotide diversity (Supplementary Table S3). Indian desert breeds such as Bikaneri, Jaisalmeri, Jalori, Kutchi, Malvi, Marwari, Mewari, and Sindhi showed relatively fewer variable sites (19–26) and low nucleotide diversity (π ≈ 0.0028–0.0032) compared to other dromedary sequences. This diversity corresponds to an average of only 3–4 nucleotide differences (k) between any two sequences, suggesting comparatively low genetic divergence among Indian camel haplotypes. Such patterns correlate with long-term and restricted maternal lineage diversification within Indian desert populations. In contrast, the Arabian Peninsula and Iranian populations exhibited substantially higher levels of polymorphism. Both groups showed a greater number of segregating sites (28–30), and elevated nucleotide diversity (π ≈ 0.0041–0.0044), corresponding to an average of 5 haplotype (k) per sequence pair. This suggests that dromedaries from the Arabian Peninsula and Iran exhibit deeper haplotype divergence and greater mitochondrial diversity, which may be attributed to either larger effective population sizes, multiple maternal lineages, or historical demographic expansion.

The haplotype network based on D-loop sequences revealed distinct patterns between Indian and other camel breeds (Supplementary Table S3 and Figure S2). For Indian breeds, most haplotypes appeared as unique, population-specific nodes of equal size. This produced very high haplotype diversity (Hd = 1.0) but relatively low nucleotide diversity (π ≈ 0.0030; k = 3–4), suggesting limited sequence divergence. By contrast, the Arabian Peninsula and Iranian populations exhibited larger nodes with multiple individuals sharing haplotypes and more complex branching patterns, consistent with higher nucleotide diversity (π > 0.004; k ≈ 5) and deeper lineage divergence.

### 3.7. Functional Assessment of D-Loop Sequence

Transcription factor binding site analysis of the D-loop region in the dromedary mitochondrial genome revealed several high-confidence binding motifs distributed across the breeds. Predicted sites exhibited relative scores ranging from 0.78 to 0.89, indicating a substantial likelihood of functional regulatory activity within this region. Notably, recurrent motifs such as “tgtGGGGGTTTCTata” and “gcaGGGATCCCTCttc” were identified at positions 287 and 322 bp, consistently observed across Bikaneri, Kharai, Malvi, Jalori, Marwari, Sindhi, and Mewari breeds. These motifs displayed robust statistical support, with *p*-values less than 0.001 for each prediction.

## 4. Discussion

The D-loop region of the mitochondrial genome can be used to describe evolutionary processes and taxonomic classification of domestic animal species. Unlike the coding region, the D-loop is a non-coding control sequence present in the mitochondrial genome. It plays a critical role in replication and transcriptional initiation, making it an ideal candidate to explore evolutionary events at the structural and regulatory levels [1]. A comprehensive analysis of the mitochondrial D-loop region for Indian dromedary breeds was conducted, with special emphasis on phylogenetic relationships, compositional patterns, diversity indices, and population structure. In addition, multivariate analyses, were explored to complement the haplotype-based polymorphism assessment. The analysis of camel population for polymorphism in haplotype-based diversity, gene-based phylogenies, or SNP-based comparisons in the mitochondrial genome has been reported in previous studies [14,18]. However, these approaches cannot capture spatial and compositional biases, essential for understanding replication fidelity, evolutionary constraints, and the functional architecture of the D-loop sequence.

The overall findings reflect low mitochondrial genomic divergence among Indian dromedary breeds, indicating a highly conserved D-loop region (~99.4%). This limited variability could be attributed to shared evolutionary history, narrow mitochondrial genetic base, limited lineage diversification, or historical gene flow among Indian dromedary camel populations. The close clustering of Indian dromedary breeds in the MCC tree can be corroborated to earlier studies that suggested weak phylogeographic structuring within Indian dromedary populations [18]. In contrast, Iranian and Arabian Peninsula camels formed distinct clusters, underscoring clear lineage separation and higher divergence, consistent with earlier mitogenomic investigations [10,11,13,14,30].

The nucleotide composition of the D-loop exhibited conservation across Indian dromedaries, characterized by a stable sequence length and an AT-rich profile. Such compositional biases were already described for mitochondrial control regions of other livestock species including cattle, sheep, and goats [3,7]. The enriched CpG motifs within the D-loop sequence, compared to the complete mitochondrial genome, suggest potential functional conservation of regulatory sequences [5,31]. Correlation and correspondence analyses of D-loop sequences further stressed the non-random organization and conserved usage of nucleotides. These rigorous nucleotide distribution patterns ensure strand asymmetry and replication-associated mutational pressures, a phenomenon previously reported in sheep and other ruminants [8,9,16]. The significant association of AT-richness with homopolymeric adenine repeats in the D-loop suggests tolerance for sequence plasticity, which may facilitate the formation of secondary structures critical for regulatory function [6].

Population structure analysis estimated that nearly all mitochondrial variation was distributed between breeds, with negligible within-breed variation. Pairwise genetic distance confirmed high divergence between Iranian/Arabian Peninsula and Indian dromedaries, while Indian desert breeds such as Bikaneri, Jaisalmeri, Marwari, Jalori, and Mewari displayed minimal differentiation. Coastal and peripheral breeds of India, such as Kharai, Sindhi, and Malvi, exhibited moderate divergence. These results indicate that Indian dromedaries are genetically homogeneous but distinct from foreign populations, certifying limited lineage diversification. Neutrality analyses provided further resolution on population demography. Population-wise, Arabian Peninsula, Iranian, Sindhi, and Kharai camels exhibited significantly negative Tajima’s D score, supporting population expansion or post-bottleneck recovery. Conversely, desert Indian breeds showed values close to neutrality, indicating population stability. Such stability may suggest long-term equilibrium or restricted ancestral lineage diversification (π ≈ 0.003; k = 3–4). By contrast, Iranian and Arabian populations harbored higher polymorphism within population (π ≈ 0.004–0.0044; k ≈ 5), suggesting deeper haplotype divergence and larger effective population sizes [10,11].

The functional role of the D-loop nucleotide sequence is supported by the presence of predicted transcription factor binding motifs, consistently retained across Indian dromedaries with significant statistical support. These D-loop DNA conserved motifs likely play regulatory roles in replication initiation, further reinforcing the role of purifying selection in the mitochondrial genome [15,16]. Such structural and functional stability is essential for maintaining mitochondrial genome integrity and is reported across *Camelus* species [17].

Incidentally, the findings of Bahbahani, Al-Zoubi, Ali, Afana, Dashti, Al-Ateeqi, Wragg, Al-Bustan and Almathen [10] and Mohandesan, Fitak, Corander, Yadamsuren, Chuluunbat, Abdelhadi, Raziq, Nagy, Stalder and Walzer [11], based on the whole mitogenome of *Camelus* species, provide a broader context for interpreting the present study. Both of these studies emphasized the pervasive influence of purifying selection in maintaining mitochondrial genomic integrity. The present study complements the broader investigations by highlighting conserved sequence features and the limited variability of the D-loop among Indian dromedary breeds. The pronounced mitochondrial similarity within Indian dromedaries likely reflects a combination of evolutionary forces, including strong purifying selection to maintain functional stability, coupled with limited breed differentiation, historical breeding practices, ongoing gene flow, and a restricted geographic distribution.

The comparison of these findings with other domestic livestock species showed the mitochondrial homogeneity in Indian camels is reminiscent of similar patterns reported in *Bos indicus* populations within India [32]. In contrast, ovine and caprine species show more extensive mitochondrial lineage differentiation, often associated with multiple domestication events and the influence of geographical barriers [7]. Notably, geographical proximity does not necessarily preclude lineage-specific divergence, as demonstrated in wild sheep (*Ovis vignei*), wherein distinct D-loop variation and lineage separation have been observed despite close spatial distribution [8]. The absence of comparable fine-scale divergence in Indian dromedary breeds therefore points to long-term continuity in breeding practices and limited lineage diversification.

These patterns also underline broader demographic trends in camelids worldwide. While many countries in Africa, the Arabian Peninsula, and Asia report growing camel populations, India continues to experience a steady decline [33]. A genetically uniform population, such as the Indian dromedary, may become inherently more vulnerable to environmental calamity, emerging diseases, and reduced reproductive fitness [19]. The observed mitochondrial homogeneity can also increase the risk of loss of the adaptive potential under climate change and epidemiological pressures. Although females primarily mediate mitochondrial inheritance, in rare scenarios males can be equally crucial for mitochondrial gene flow in progeny populations [4] and need to be included for effective conservation planning and genetic resource management. This is particularly relevant given the declining camel population in India and the ecological and economic importance of camels in arid regions [33]. Preservation of existing genetic variation, while exploring strategies to manage genome diversity, is therefore critical for long-term sustainability.

## 5. Conclusions

This study offers a novel perspective on utilizing non-coding regions, such as the mitochondrial D-loop, for interpreting population genetic structure. It also presents a compositional assessment of nucleotide sequences to complement analyses of functional properties, sequence homogeneity and haplotype divergence within the dataset. Although only a small region of the mitochondrial genome was analyzed for the overall population comparison, the results clearly demonstrate that Indian dromedary breeds possess highly conserved mitochondrial D-loop sequences. In contrast, populations form Iran and the Arabian Peninsula exhibit greater genetic variability, lineage divergence, and signs of population expansion. Therefore, the mitochondrial genome, as an evolutionary imprint, provides valuable insights into the demographic dynamics of dromedaries in India. This approach can be further extended to other camelids to better understand their evolutionary patterns.

## Figures and Tables

**Table 1:** Between-breed identity values for mitochondrial D-loop nucleotide sequence in Indian dromedary camels (Mean ± SD).

Camel Breed	Bikaneri	Jaisalmeri	Jalori	Kharai	Kutchi	Malvi	Marwari	Mewari
Intra-breed	99.6 ± 0.44	99.68 ± 0.18	99.61 ± 0.38	99.88 ± 0.1	99.76 ± 0.17	99.32 ± 0.58	99.85 ± 0.11	99.9 ± 0.11
Jaisalmeri	99.66 ± 0.35							
Jalori	99.69 ± 0.38	99.67 ± 0.32						
Kharai	99.7 ± 0.34	99.77 ± 0.14	99.7 ± 0.3					
Kutchi	99.67 ± 0.36	99.74 ± 0.19	99.7 ± 0.34	99.76 ± 0.14				
Malvi	99.5 ± 0.53	99.52 ± 0.46	99.49 ± 0.51	99.55 ± 0.44	99.53 ± 0.46			
Marwari	99.73 ± 0.35	99.78 ± 0.17	99.75 ± 0.32	99.82 ± 0.11	99.82 ± 0.15	99.6 ± 0.43		
Mewari	99.76 ± 0.36	99.81 ± 0.17	99.78 ± 0.32	99.85 ± 0.11	99.85 ± 0.14	99.62 ± 0.46	99.9 ± 0.11	
Sindhi	99.77 ± 0.35	99.8 ± 0.16	99.77 ± 0.32	99.85 ± 0.11	99.83 ± 0.14	99.62 ± 0.45	99.89 ± 0.11	99.92 ± 0.11

**Table 2:** Nucleotide composition of D-loop and mitochondrial genome of Indian dromedary breeds. The percent nucleotide composition indicated as Mean ± SD.

Breed	D-loop							Mitochondrial Genome						
	Length (nt)	% A	% T	% C	% G	% A + T	% G + C	Length (nt)	%A	%C	%T	%G	%A + T	%G + C
Bikaneri	1213	29.72 ± 0.08	23.62 ± 0.11	29.33 ± 0.1	17.33 ± 0.08	53.34 ± 0.07	46.66 ± 0.07	16,643	30.83 ± 0.03	26.57 ± 0.02	27.1 ± 0.02	15.49 ± 0.03	57.94 ± 0.01	42.06 ± 0.01
Jaisalmeri	1213	29.68 ± 0	23.71 ± 0.15	29.3 ± 0.12	17.31 ± 0.06	53.39 ± 0.15	46.61 ± 0.15	16,643	30.83 ± 0.01	26.57 ± 0.02	27.11 ± 0.02	15.49 ± 0.01	57.94 ± 0.02	42.06 ± 0.02
Jalori	1213	29.71 ± 0.13	23.36 ± 0.19	29.68 ± 0.29	17.25 ± 0.11	53.08 ± 0.25	46.92 ± 0.25	16,643	30.83 ± 0.03	26.6 ± 0.02	27.08 ± 0.01	15.49 ± 0.03	57.91 ± 0.03	42.09 ± 0.03
Kharai	1211–1213	29.67 ± 0.01	23.6 ± 0.27	29.37 ± 0.23	17.35 ± 0.04	53.27 ± 0.27	46.73 ± 0.27	16,642–16,643	30.83 ± 0.01	26.57 ± 0.02	27.11 ± 0.02	15.49 ± 0.01	57.94 ± 0.02	42.06 ± 0.02
Kutchi	1213	29.66 ± 0.07	23.61 ± 0.22	29.41 ± 0.32	17.31 ± 0.17	53.27 ± 0.19	46.73 ± 0.19	16,643	30.82 ± 0.01	26.58 ± 0.02	27.11 ± 0.02	15.5 ± 0.01	57.93 ± 0.02	42.07 ± 0.02
Malvi	1207–1213	29.68 ± 0.1	23.41 ± 0.08	29.68 ± 0.11	17.23 ± 0.09	53.11 ± 0.07	46.89 ± 0.07	16,638–16,643	30.83 ± 0.02	26.58 ± 0.03	27.1 ± 0.02	15.49 ± 0.03	57.93 ± 0.04	42.07 ± 0.04
Marwari	1213	29.65 ± 0.04	23.33 ± 0.1	29.74 ± 0.35	17.28 ± 0.22	52.98 ± 0.14	47.02 ± 0.14	16,643	30.83 ± 0.02	26.61 ± 0.03	27.08 ± 0.01	15.48 ± 0.02	57.91 ± 0.02	42.09 ± 0.02
Mewari	1213	29.66 ± 0.04	23.33 ± 0.16	29.78 ± 0.19	17.23 ± 0.07	52.99 ± 0.18	47.01 ± 0.18	16,643	30.84 ± 0.03	26.61 ± 0.02	27.08 ± 0.02	15.48 ± 0.02	57.92 ± 0.02	42.08 ± 0.02
Sindhi	1213	29.66 ± 0.04	23.43 ± 0.35	29.73 ± 0.5	17.18 ± 0.17	53.09 ± 0.33	46.91 ± 0.33	16,643	30.83 ± 0	26.6 ± 0.04	27.09 ± 0.02	15.48 ± 0.01	57.92 ± 0.02	42.08 ± 0.02
Overall	1207–1213	29.68 ± 0.06	23.49 ± 0.18	29.56 ± 0.25	17.27 ± 0.11	53.17 ± 0.18	46.83 ± 0.18	16,638–16,643	30.83 ± 0.02	26.59 ± 0.02	27.1 ± 0.02	15.49 ± 0.02	57.93 ± 0.02	42.07 ± 0.02

**Table 3:** Correlation analysis of nucleotide composition parameters for camelid mitochondrial D-loop region.

Correlation	A	T	C	G	AT%	GC%	A-DD	C-DD	G-DD	T-DD	NR-A	NR-C	NR-G	NR-T
T	−0.792 **	1												
C	0.793 **	−0.955 **	1											
G	−0.795 **	0.420 **	−0.593 **	1										
AT%	−0.412 **	0.883 **	−0.814 **	0.015	1									
GC%	0.412 **	−0.883 **	0.814 **	−0.015	−1.000 **	1								
A-DD	−0.702 **	0.955 **	−0.861 **	0.254 *	0.886 **	−0.886 **	1							
C-DD	−0.545 **	0.849 **	−0.729 **	0.081	0.847 **	−0.847 **	0.907 **	1						
G-DD	0.222	0.303 *	−0.09	−0.710 **	0.623 **	−0.623 **	0.463 **	0.545 **	1					
T-DD	0.460 **	−0.573 **	0.542 **	−0.238	−0.501 **	0.501 **	−0.443 **	−0.377 **	−0.101	1				
NR-A	−0.103	0.628 **	−0.565 **	−0.218	0.858 **	−0.858 **	0.740 **	0.774 **	0.700 **	−0.171	1			
NR-C	0.389 **	0.178	−0.006	−0.773 **	0.565 **	−0.565 **	0.317 *	0.487 **	0.923 **	−0.042	0.722 **	1		
NR-G	−0.675 **	0.905 **	−0.771 **	0.175	0.831 **	−0.831 **	0.958 **	0.954 **	0.504 **	−0.418 **	0.700 **	0.367 **	1	
NR-T	−0.1	0.509 **	−0.533 **	−0.028	0.682 **	−0.682 **	0.603 **	0.580 **	0.395 **	−0.032	0.905 **	0.474 **	0.524 **	1

** Significance at the 0.01 level; * Significance at the 0.05 level; DD: distance distribution; NR: nucleotide repeats.

## Data Availability

All the data used and generated are presented along the manuscript.

## Supplementary Materials

The following supporting information can be downloaded at https://www.mdpi.com/article/10.3390/ani15213070/s1. Figure S1: Hierarchical clustering dendrogram of the dromedary D-loop sequence dataset based on genetic distance. Figure S2: Haplotype median joining network analysis for dromedary D-loop sequence dataset. Figure S3: Prediction of transcription factor binding sites for dromedary D-loop sequence dataset. Table S1: Sequence dataset used. Table S2: Population structure analysis. Table S3: Analysis of haplotype diversity.

## Abbreviations

The following abbreviations are used in this manuscript:

mtDNA	Mitochondrial DNA
D-loop	Displacement loop
ATP6	Adenosine Triphosphate Synthase subunit 6
ATP8	Adenosine Triphosphate Synthase subunit 8
SNP	Single Nucleotide Polymorphism
SAS	Statistical Analysis System
ME	Minimum Evolution
CNI	Close-Neighbor Interchange
A	Adenine
T	Tyrosine
G	Guanine
C	Cytosine
nt	Nucleotides
SD	Standard Deviation
O/E	Observed/Expected
r	Pearson correlation coefficient
A-DD	Adenine Distance Distribution
AT%	Adenine–Thymine percentage
