# Supplementary material for: Phylogenetic Analysis of Indian Dromedary Breeds Based on the Mitochondrial D-Loop Marker
Source: Animals (Basel). 2025 Oct 23;15(21):3070. doi: 10.3390/ani15213070 (PMC12610032; doi:10.3390/ani15213070)

Figure S3: Prediction of transcription factor binding sites for dromedary D-loop sequence dataset

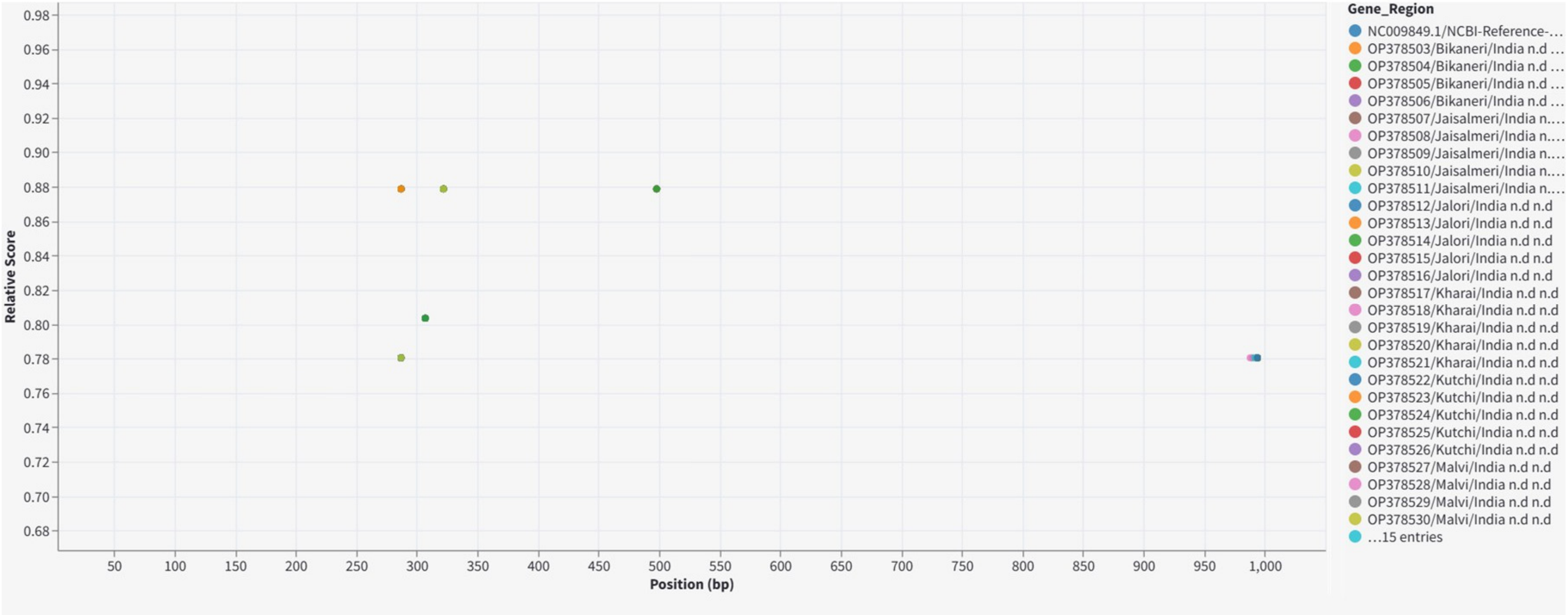

Supplement: Supplementary file 1 [file animals-15-03070-s001.zip › Figure S3.pdf]
