# Supplementary material for: Phylogenetic Analysis of Indian Dromedary Breeds Based on the Mitochondrial D-Loop Marker
Source: Animals (Basel). 2025 Oct 23;15(21):3070. doi: 10.3390/ani15213070 (PMC12610032; doi:10.3390/ani15213070)
Supplement: Supplementary file 1 [file animals-15-03070-s001.zip › Table S1.pdf]

Table S1: Sequence dataset used

Species	Sequence submitted from Country	Particular (Breed/ Animal Id/ etc.)	GenBank ® Accession number
**Dromedary**	India	Bikaneri	OP378503
**Dromedary**	India	Bikaneri	OP378504
**Dromedary**	India	Bikaneri	OP378505
**Dromedary**	India	Bikaneri	OP378506
**Dromedary**	India	Jaisalmeri	OP378507
**Dromedary**	India	Jaisalmeri	OP378508
**Dromedary**	India	Jaisalmeri	OP378509
**Dromedary**	India	Jaisalmeri	OP378510
**Dromedary**	India	Jaisalmeri	OP378511
**Dromedary**	India	Jalori	OP378512
**Dromedary**	India	Jalori	OP378513
**Dromedary**	India	Jalori	OP378514
**Dromedary**	India	Jalori	OP378515
**Dromedary**	India	Jalori	OP378516
**Dromedary**	India	Kharai	OP378517
**Dromedary**	India	Kharai	OP378518
**Dromedary**	India	Kharai	OP378519
**Dromedary**	India	Kharai	OP378520
**Dromedary**	India	Kharai	OP378521
**Dromedary**	India	Kutchi	OP378522
**Dromedary**	India	Kutchi	OP378523
**Dromedary**	India	Kutchi	OP378524
**Dromedary**	India	Kutchi	OP378525
**Dromedary**	India	Kutchi	OP378526
**Dromedary**	India	Malvi	OP378527
**Dromedary**	India	Malvi	OP378528
**Dromedary**	India	Malvi	OP378529
**Dromedary**	India	Malvi	OP378530
**Dromedary**	India	Malvi	OP378531
**Dromedary**	India	Marwari	OP378532
**Dromedary**	India	Marwari	OP378533
**Dromedary**	India	Marwari	OP378534
**Dromedary**	India	Marwari	OP378535
**Dromedary**	India	Marwari	OP378536
**Dromedary**	India	Mewari	OP378537
**Dromedary**	India	Mewari	OP378538
**Dromedary**	India	Mewari	OP378539
**Dromedary**	India	Mewari	OP378540
**Dromedary**	India	Sindhi	OP378542
**Dromedary**	India	Sindhi	OP378543
**Dromedary**	India	Sindhi	OP378544
**Dromedary**	India	Sindhi	OP378545
**Dromedary**	India	Sindhi	OP378546
**Dromedary**	Qatar	AlMzayen	KU605072

**Dromedary**	Saudi Arabia	Magaheem	KU605073
**Dromedary**	Saudi Arabia	Homor	KU605074
**Dromedary**	Saudi Arabia	Wadda	KU605075
**Dromedary**	Iran	Yazdi	KX554931
**Dromedary**	Iran	Kalkuei	KX554932
**Dromedary**	Iran	Dashti	KX554934
**Dromedary**	Saudi Arabia	NCBI reference	NC009894
**Wild camel**	China	NCBI reference	NC009629
