# Supplementary material for: Phylogenetic Analysis of Indian Dromedary Breeds Based on the Mitochondrial D-Loop Marker
Source: Animals (Basel). 2025 Oct 23;15(21):3070. doi: 10.3390/ani15213070 (PMC12610032; doi:10.3390/ani15213070)
Supplement: Supplementary file 1 [file animals-15-03070-s001.zip › Table S2.pdf]

Table S2: Population structure analysis

Parameter	Bikaneri	Jaisalmeri	Jalori	Kharai	Kutchi	Malvi	Marwari	Mewari	Sindhi	Arabian Peninsula	Iranian
Sample size (N)	4	5	3	4	3	4	4	5	4	5	5
Sum of squared deviations (SSD)	0.0021 (0.68)	0.0019 (0.70)	0.0023 (0.65)	0.0030 (0.62)	0.0028 (0.64)	0.0025 (0.66)	0.0022 (0.67)	0.0026 (0.65)	0.0029 (0.63)	0.0064 (0.51)	0.0099 (0.51)
Tajima's D (p-value)	-0.12 (0.54)	-0.08 (0.57)	-0.10 (0.55)	-0.15 (0.52)	-0.11 (0.53)	-0.09 (0.56)	-0.14 (0.53)	-0.07 (0.57)	-0.13 (0.52)	-1.23 (0.08)	-0.94 (0.20)
Fu's Fs	-2.1	-1.85	-2	-2.5	-2.2	-2.05	-2.3	-1.9	-2.4	-17.16	-2.83
