# Supplementary material for: Phylogenetic Analysis of Indian Dromedary Breeds Based on the Mitochondrial D-Loop Marker
Source: Animals (Basel). 2025 Oct 23;15(21):3070. doi: 10.3390/ani15213070 (PMC12610032; doi:10.3390/ani15213070)
Supplement: Supplementary file 1 [file animals-15-03070-s001.zip › Table S3.pdf]

Table S3: Analysis of haplotype diversity

Population	Number of individuals	Number of haplotypes	Number of variable sites (S)	Haplotype Diversity (Hd)	Nucleotide Diversity (pi)	Average number of nucleotide differences (k)
Bikaneri	4	4	23	1	0.003217	4.0315
Jaisalmeri	5	5	25	1	0.003512	4.409
Jalori	3	3	19	1	0.00277	3.4698
Kharai	4	4	22	1	0.003073	3.8478
Kutchi	3	3	26	1	0.003915	4.902
Malvi	4	4	21	1	0.003158	3.9586
Marwari	4	4	20	1	0.003001	3.7656
Mewari	5	5	24	1	0.00341	4.2685
Sindhi	4	4	23	1	0.003217	4.0315
Arabian Peninsula	5	5	30	1	0.00436	5.458
Iranian	5	5	28	1	0.004083	5.116
